# Video Content Analysis of Human Sports under Engineering Management Incorporating High-Level Semantic Recognition Models

**DOI:** 10.1155/2022/6761857

**Published:** 2022-01-12

**Authors:** Ruan Hui

**Affiliations:** Shanxi University, Shanxi, Taiyuan 030006, China

## Abstract

In this paper, a high-level semantic recognition model is used to parse the video content of human sports under engineering management, and the stream shape of the previous layer is embedded in the convolutional operation of the next layer, so that each layer of the convolutional neural network can effectively maintain the stream structure of the previous layer, thus obtaining a video image feature representation that can reflect the image nearest neighbor relationship and association features. The method is applied to image classification, and the experimental results show that the method can extract image features more effectively, thus improving the accuracy of feature classification. Since fine-grained actions usually share a very high similarity in phenotypes and motion patterns, with only minor differences in local regions, inspired by the human visual system, this paper proposes integrating visual attention mechanisms into the fine-grained action feature extraction process to extract features for cues. Taking the problem as the guide, we formulate the athlete's tacit knowledge management strategy and select the distinctive freestyle aerial skills national team as the object of empirical analysis, compose a more scientific and organization-specific tacit knowledge management program, exert influence on the members in the implementation, and revise to form a tacit knowledge management implementation program with certain promotion value. Group behavior can be identified by analyzing the behavior of individuals and the interaction information between individuals. Individual interactions in a group can be represented by individual representations, and the relationship between individual behaviors can be analyzed by modeling the relationship between individual representations. The performance improvement of the method on mismatched datasets is comparable between the long-short time network based on temporal information and the language recognition method with high-level semantic embedding vectors, with the two methods improving about 12.6% and 23.0%, respectively, compared with the method using the original model and with the *i*-vector baseline system based on the support vector machine classification method with radial basis functions, with performance improvements about 10.10% and 10.88%, respectively.

## 1. Introduction

With the continuous development of information technology, the way people obtain and store massive video information keeps developing towards diversification, and video information gradually becomes the mainstream multimedia data carrier. In the context of huge video data resources, users face the challenge of how they can efficiently retrieve video resources according to their interests [1]. Therefore, it is necessary to classify and organize the massive video resources intelligently to facilitate users to retrieve according to their preferences. Video semantic analysis technology can annotate and classify important semantic information in videos, and users can retrieve it according to their preferred categories, which improves the efficiency of users' access to information. In addition, the host-based implementation of video semantic analysis technology can replace manual annotation work, reducing many human resources and improving information utilization. Video semantic concept analysis refers to the generalized description of video content after obtaining video sequences, and the content of events, scenes, objects, and so forth is the multicategory semantic information contained in semantic concepts. A large amount of video data has large intraclass variations for the same action class, which may be caused by background clutter, viewpoint changes, and movement speed and style [2]. In particular, the feature information generated by the deep learning model is very large. Only when the attention mechanism is used in the huge feature space can the model extract more effective features and discard useless information. The high dimensionality and low resolution of the videos further increase the difficulty of designing efficient and robust recognition methods. Although traditional manual annotation methods can achieve the understanding and description of video semantic concepts to a certain extent, the time and labor cost of manual annotation are huge, subjective, and difficult to cross the semantic gap between the underlying features and the semantic understanding of video data, and its annotation speed cannot achieve efficient classification and organization of video data [3]. Therefore, in recent years, researchers have focused their research on how they can automatically access the semantic concepts of video data and annotate, classify, and organize rich video data. This research has significant academic and applied value and helps to improve video management techniques to make them more complete and more efficient.

Video semantic concept analysis is a key and difficult area in the field of machine learning and pattern recognition, where video data can be efficiently and intelligently retrieved and organized by recognizing and understanding the main events, scenes, and objects in the video. In recent years, with the rapid development of technology and the improvement of the computing power of hardware devices, the way of video acquisition has become faster [4, 5]. Video retrieval is the process of finding a match in the video database according to the user's textual description according to a specific algorithm and filtering all videos that match the user's needs according to some qualifying conditions. Group behavior recognition technology can derive labels for crowd scene images that can provide clues for retrieval of images and videos of group scenes. The advancement of group behavior recognition technology is a great boost to crowd scene classification, labeling, and retrieval. With the popularity of electronic devices, especially mobile electronic devices such as mobile phones, the change in people's lifestyles, and the need to record their productive lives, a large amount of image and video data has been generated. However, it is not a simple task to manage and utilize these huge amounts of images and data [6]. A good starting point can not only prevent the gradient descent algorithm from falling into the local extreme point which is difficult to jump out of but also reduce the time to find the global optimal solution, if the initialization point is close enough to the optimal solution. Current video retrieval technology relies heavily on users submitting and sharing videos along with video subject descriptions. This is difficult to achieve in real time, and it is difficult to get down to the specifics, as describing specific details is tedious and time-consuming, and only based on image and video analysis techniques can achieve real-time frame-by-frame analysis. Group behavior recognition techniques are of great interest for real-time frame-level video classification retrieval of crowd scenes.

Classification and detection of video-based actions is an important research topic in the field of computer vision, which has a very wide range of applications in intelligent human-computer interaction, video surveillance, telemedicine, and other fields. The difficulties of traditional action analysis tasks mainly come from several aspects: the differences arising from the same action performed by different people; the influence of environmental factors, such as occlusion, viewpoint changes, lighting differences, and dynamic background interference; and the ambiguity in defining the starting and ending points of the action. However, the existing recognition and detection performance still falls far short of the requirements for accuracy in practical applications. The reason for this is that, on the one hand, the number of action classes in the existing dataset is limited and cannot cover all actions in realistic scenes, and the definition of the classes is relatively coarse, so the models trained on the coarse-scale action classes are not able to analyze increasingly fine-grained action classes. Another important reason is that action classes in existing datasets are usually hand-selected and cropped, resulting in more significant episodic and motion differences between classes, while action boundaries in realistic scenes are usually fuzzy and uncertain, and similarities between actions are often large, often with only minor local differences in the actions. In such cases, more fine-grained action discrimination and detection are required. Therefore, we believe that fine-grained action analysis will facilitate further breakthroughs in the task of action classification and detection, thus promoting the advancement of abstract theoretical research to practical applications in realistic scenes.

## 2. Related Work

How to represent the behavior in the video is the core problem of behavior recognition research, which determines the recognition performance to a certain extent. There exist many feature representation methods, which can be divided into two categories according to the source of features: handcrafted features and features learned from samples [7]. Handcrafted features are features designed by research experts based on human visual principles. In contrast, features obtained from samples are learned without prior feature design, and suitable feature representations are found directly in training samples by various types of learning algorithms, among which deep learning methods have become the mainstream method for learning features due to their excellent performance [8]. It is pointed out that implicit knowledge is real in the practice of competitive sports, especially in the acquisition of motor skills where implicit cognition plays an important role, and a coping strategy is proposed on how implicit cognition and explicit cognition can be transformed into each other in motor skill learning. Large-scale image retrieval systems necessarily have high demands on the time overhead of retrieval [9]. The performance problem is a difficult problem that must be solved for retrieval on a collection of images of hundreds of millions of sizes [10]. The basis of image retrieval lies in the similarity calculation between the query image and the database image in the feature space. Calculating the similarity between each vector following a linear traversal is very time-consuming [11].

How to ensure the efficiency of image retrieval systems has been a key research direction in the fields of information retrieval, machine learning, and computer vision [12]. Considering that the image features to be retrieved are not uniformly diffuse in the feature space but in some specific distribution pattern, it is sometimes not necessary to traverse the whole query space to query for the nearest neighbor feature vector. Based on this idea, many kinds of tree-based index structures were proposed to narrow the retrieval domain of the query vector by recursively partitioning the feature space. The vector quantization approach, on the other hand, lies in approximating the original features using quantization to certain representative elements [13]. Deep learning methods can learn multilayer feature hierarchies and automatically construct high-level representations of the original input. A large amount of data is used to drive network training and model optimization to extract more representative semantic features, thereby improving classification performance, making the limitations of traditional manually designed feature extraction methods largely avoided. Using quantization methods, the global features of an image are usually quantized to sparse storage, and the query features are associated with only a small number of relevant quantization points during the similarity calculation, which corresponds to a reduction in the dimensionality of the image features and therefore an increase in retrieval speed [14].

In terms of tacit knowledge measurement methods, most of the relevant results of hierarchical analysis and fuzzy integrated measurement methods are used for specific applications [15]. In recent years, many scholars have started to try to combine the relevant theories of discrete mathematics with multicriteria decision-making to characterize the weight information for multiple scenario comparisons and decision-making. The theory of partial order set, as an important element in discrete mathematical order theory, is a very attractive decision support tool, and the options can be compared and ranked by qualitative weight information. No systematic theoretical system has been developed. Many scholars have addressed the influence of nontechnical factors on athletes' performance from a psychological and philosophical perspective. There are more discussions about implicit learning in psychology, alienation, and sustainable development of competitive sports from a philosophical perspective. The literature applying knowledge management theory to the field of competitive sports practice is occasionally found to be more general. A systematic study of the basic theoretical issues of athletes' tacit knowledge will certainly make up for the lack of research in this field to a certain extent, thus promoting the enrichment and development of the theoretical system of athletes' tacit knowledge.

## 3. Video Content Analysis of High-Level Semantic Recognition Model Sports under Engineering Management

### 3.1. High-Level Semantic Recognition Model Design under Engineering Management

Interaction information is an important clue for the task of group behavior identification, and mining the interaction information between individuals is crucial for identifying individual behavior and group behavior. Group behavior can be identified by analyzing the behavior of individuals and the interaction information between individuals. Individual interactions in a cluster can be represented by individual representations, and analysis of the relationships between individual behaviors can be modeled by modeling the relationships between individual representations [16]. It is necessary to extract the appearance representation of individuals, to establish the relationship between individual interaction and individual representation, to analyze the behavior of individuals and to analyze the model of group behavior and individual interaction, and to obtain the group behavior by analyzing individual representation, individual behavior, and interaction between individuals. The attention mechanism is used in the individual fusion of interaction information. After completing the establishment of the index based on the random split rule, we input the automatically mined dance element into each random tree in turn and implement the top-down matching process to find its nearest neighbor in the feature space. By attention mechanism, we mean that the model devotes more attention to the important information and allocates more attention to it. The attention mechanism is essentially designed to pick out the most representative information and discard features with less information. In particular, the feature information generated by deep learning models is very large, and only by using the attention mechanism in large feature space can the model extract more effective features and discard useless information. Attention mechanisms can be used on different dimensions of features, spatial attention to determine which region in the image is more significant and should receive attention, temporal attention to determine which moment in the temporal sequence contains more information, and channel attention to highlight the important role of certain channels in the feature. Achieving attention can be done in both hard and soft ways; the hard attention approach is by completely retaining and completely discarding some information. The soft attention approach generates new states by calculating the weight of information in the new state. Through this form, it is possible to discover the cognitive differences between the two knowledge elements that directly affect the sports performance of the snow sports athletes to provide effective help for coaches to adopt targeted training methods and guidance strategies.

(1) $$\begin{array}{c} \max\phi \left(w\right)=\frac{1}{2}\left(w_{1}\cdot w^{2}\right)\\ \text{subject to}y_{i}\left[\left(w\cdot x_{i}\right)-b\right]-2\geq 0,\;i\in N, \end{array}$$

where *x*_*i*_ and *y*_*i*_^2^ represent the *i*-vector of the *i*-th training language sample and the corresponding labels, respectively, and *w* and *b* are the parameters of the classification hyperplane to be trained. This is an optimization problem with constraints, and thus it can be optimized using the Lagrange multiplier method, so the following function is defined:

(2) $$\begin{array}{c} L\left(w,b,\alpha \right)=\frac{1}{2}\left(w\cdot w^{2}\right)-\sum_{i=1}^{n}\alpha_{i}\left\{y_{i}^{2}\left[w\cdot x\right]-b\right\}. \end{array}$$

**Figure 1:**
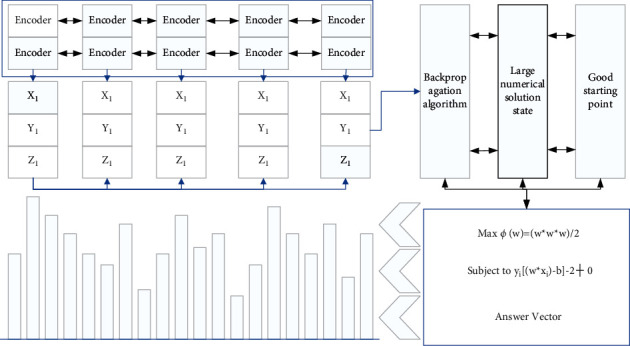
High-level semantic recognition model.

Since the error back-propagation algorithm is, in fact, a search for states close to the extremum in a large numerical solution state space, a good starting point can both prevent the gradient descent algorithm from getting stuck in local extremes that are difficult to jump out of and reduce the time to find the global optimal solution, if the initialization point is close enough to the optimal solution. Moreover, the response threshold of the activation function is finite due to the nonlinear factor of the model [17]. A good initialization parameter can reasonably activate the activation function so that most parameters are involved in the training, allowing most neurons to participate in the expression without dying, as shown in Figure 1.

The data distribution of the dataset is often fixed and we cannot change the data distribution of the dataset, so the distribution of the parameters directly affects the output response of this network, and if the range of the region of the response is not in the expected interval, then the loss of the model is huge and hard to debug. The parameter-transformed output data should not appear in some uncommon zones, which will make it more difficult to fit the model and reduce its capacity.

(3) $$\begin{array}{c} \left\{\begin{array}{c} \max{\left\|\alpha_{i}\right\|}_{1}\;\text{s.t. }x_{i}=X\alpha_{i}^{2},\\ I^{T}\alpha_{i}=1. \end{array}\right. \end{array}$$

The conceptual analysis of video semantics has been a more active research direction in recent years due to the potential applications of an effective understanding of human behavior in video and its interactions in the environment in a variety of domains. To accomplish this challenging task, several research areas have worked on modeling multiple aspects of video semantics (emotions, relational attitudes, behaviors, etc). The other subset contains all the remaining video clips as the natural dataset to be recommended.

In this context, understanding the underlying semantic concepts in videos becomes crucial in interpreting complex video events. In recent years, deep learning methods have played an important role and have been widely used in computer vision tasks such as image segmentation, detection, recognition, and retrieval. In the field of video semantic concept analysis, how to cross the “semantic gap” and establish a mapping relationship between underlying features and high-level semantics to extract abstract features that are closer to the high-level semantics of video has become a core problem for researchers to solve. Deep learning methods can learn multiple layers of feature hierarchies and automatically construct high-level representations of the original input, using large amounts of data to drive the training of the network and optimization of the model to extract more representative semantic features, thus improving the classification performance, making the limitations of traditional manually designed feature extraction methods largely avoided. Since the feature construction process is fully automated, they are more general.

In our experiment, 10 video clips of each dance type are simulated to the video that the user has clicked on, and the final recommendation result is automatically obtained according to the degree of matching with the dance style excavated from the 10 input videos. Locality-sensitive discriminant analysis is a classical supervised dimensionality reduction algorithm that considers both the discriminant information in the data and the geometric structure of the data. By constructing intraclass and interclass graphs, the method can better characterize the original local features of the data manifold and preserve the original class labels of the data with good discriminability. The sparse constrained autoencoder enables the encoded learned feature representation to better obtain the sparse reconstruction relationship between data by introducing SPP-constructed graph constraints for the nonlinear autoencoder. This pretraining model not only effectively exploits the natural discriminative power of the sparse representation but also largely alleviates the difficulties in the selection of the nearest neighbor parameters.

(4) $$\begin{array}{c} J\left(f,g\right)=E\left(x,g\left(f\left(x\right)\right)\right)-\frac{1}{2}\partial t\;r\left(H^{T}G_{\text{spp}}H^{2}\right). \end{array}$$

**Figure 2:**
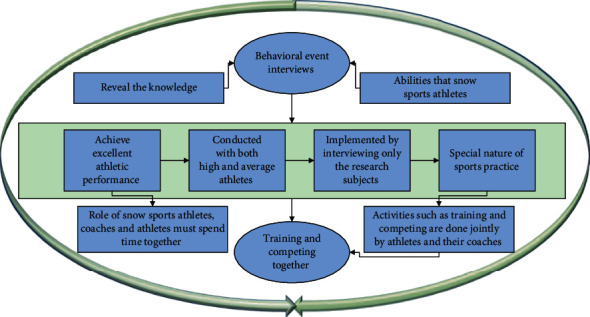
Campaign engineering management framework.

In this framework, to exploit the structural information between images, we wish to obtain the flow information of the previous layer (which can be the input or pooling layer) by constructing locality and sparsity graphs and using the flow information to redesign the mapping relationships between adjacent layers. These graph construction methods make the learned features more stable and discriminative as the network depth deepens, further speeding up the convergence and improving the generalization of the model [18]. The objective function of the localization and sparsity-preserving embedding convolutional neural network of adjacent layers consists of two components: reconstruction error between feature graphs of adjacent layers and graph regularization. After completing the establishment of the random split rule-based index, we input the automatically mined dance elements into each random tree in turn and implement a top-down matching process to find their nearest neighbors in the feature space and recommend dances in the natural dataset based on the cumulative ranking of the matches of such features, as shown in Figure 2.

Then the impact of differences like this on the performance of the language recognition model is obvious. Depending on the method of selecting spatiotemporal interest points, the current mainstream methods can be divided into spatial-temporal interest point features and trajectory features. Feature detection of local spatial-temporal feature points usually selects spatial-temporal localization and scale by maximizing a specific saliency function, and different detectors usually differ significantly in the type and sparsity of the selected points. Feature descriptors capture shape and motion features in a neighborhood of the selected point of interest using metrics such as spatial or spatial-temporal image gradients or optical flow.

(5) $$\begin{array}{c} R={\left(I^{*}g^{*}h_{gv}\right)}^{2}-{\left(I^{*}g^{*}h_{o\;d}\right)}^{2}. \end{array}$$

Behavioral event interviews, conducted with both high and average athletes in snow sports, reveal the knowledge, qualities, and abilities that snow sports athletes must have to achieve excellent athletic performance, and this is often implemented by interviewing only the research subjects themselves. However, due to the special nature of sports practice and the role of snow sports athletes, coaches and athletes must spend time together, not only training and competing together but also living together every day and “feeling and fighting” for a few years or more than ten years, and they are in contact with each other, and sports practice activities such as training and competing are done jointly by athletes and their coaches.

Considering the strong complementarity between spatial flow features and optical flow features, choosing a suitable fusion method can effectively improve the performance of video classification. This method first extracts video image frames to form image sequence and optical flow sequence. Therefore, coaches even know their strengths and weaknesses better than athletes. Based on the relevant knowledge information obtained by the snow sport athletes themselves, the behavioral event interviews with their coaches can not only provide a basis for the researcher to confirm the content elements of tacit knowledge but also find out the differences in the cognitive aspects of the knowledge elements that have a direct impact on the athletes' performance in snow sport, to provide effective help for the coaches to adopt targeted training methods and coaching strategies. This will help coaches to adopt targeted training methods and coaching strategies.

The processing of video presents more challenges compared to still images; for example, temporal sequencing is important for behavior recognition in video, but how to reflect temporal information in the representation of behavior still needs further research, as well as issues such as occlusion, background noise, and interclass differences, and further improvements in both hand-designed features and deep learning features, as well as how to fuse multiple features to improve recognition rates, require further research.

### 3.2. Experimental Design for Video Content Analysis of Sports

The information in the two hidden layers can well contain the language-related identity information of the speech segment and reflect the nature of that speech segment; that is, it can be considered as the language-related identity information of that speech segment. This representation of speech segments is more exploitable than the LSTM network model. In fact, in the traditional language recognition approach, the *i*-vector itself is also a representation of the language vector after highly abstracting the high-level semantic information, which is very similar to the nature of the embedding vector. Moreover, the *i*-vector itself assumes that the sample distribution of language recognition conforms to a Gaussian distribution, whereas LSTM networks do not have such type of assumptions [19]. Therefore, if the *i*-vector, which reflects the nature of speech segments, can be replaced by the embedding vector and then the investor-based language recognition classification method can be used for classification and scoring, theoretically, it can achieve better results than the *i*-vector method.

The subset used for dance style mining consists of 10 video segments from each dance genre; another subset contains all remaining video segments as the natural dataset to be recommended. This unbalanced method of slicing the data exactly matches the reality. We know that a user browsing a video on a website selectively selects only a small number of videos to click through, while the amount of data to be recommended in the webspace is huge. A video recommender should then be able to efficiently select videos relevant to the video content that the user has clicked on for recommendation from a large amount of distracting data. In our experiments, 10 video clips of each dance genre are simulated as videos clicked by the user, and the final recommendation results are automatically obtained based on the match with the dance styles mined from the 10 input videos.

(6) $$\begin{array}{c} \text{MAP}@N=\frac{1}{N\sum_{i=1}^{n}\left(\theta \left(r_{i}\right)/i\right)}\left(\frac{1}{n}\sum_{i=1}^{n}\frac{\theta \left(r_{i}^{2}\right)}{i_{1}^{2}}\right). \end{array}$$

Its purpose is to guide the spatial stream to pay more attention to the foreground area of the human body and reduce the influence of background noise, to better obtain the changes and differences between temporal and spatial features, and to improve the rationality of the network to extract video features. In the AP17-OLR dataset description, the dataset provider also points out that there are some differences between the training data and the test data used for the experiments, and, in the cases of Japanese, Korean, and Russian, the dataset directly gives the sampling environments between the training and test sets, both in quiet conditions and in speech segments mixed with noise. Also, the provider of the dataset points out that the sampling environments of Kazakh, Tibetan, and Uyghur are completely different from the situations of all other languages, and there are some differences between the training and test sets. Whereas DNN-like networks (including LSTMs) are more sensitive to such issues, the impact of differences like these on the performance of language recognition models is obvious if there is no good method for channel compensation, or if existing channel compensation measures are not sufficient to solve the problem. In fact, in the models described in the previous sections of this paper, there is a significant degradation in the recognition accuracy of some of the languages; taking the LSTM-1-MFCC network as an example, the false rejection and false acceptance rates for each specific language in this network are shown in Table 1.

**Figure 3:**
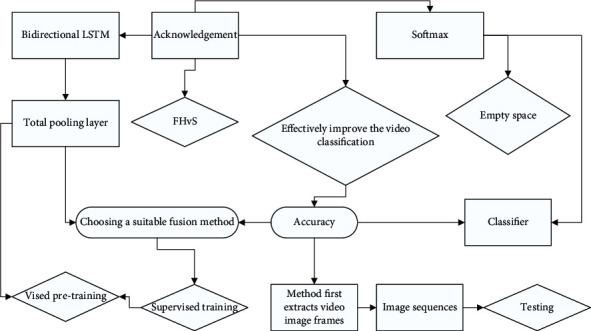
Network structure for compensating for mismatches between datasets.

The video semantic concept analysis task is richer and more complex compared to recognition tasks such as image classification, and complex situations such as background dynamic information interference, angle transformation, and target blocking can occur in different scenes. Although convolutional neural networks have achieved great success in image classification and recognition tasks, how to model the spatial-temporal features of videos and obtain the spatial-temporal information contained in videos is still one of the main problems that need to be solved urgently for video semantic concept analysis using deep learning methods. Many works have designed various effective deep convolutional neural networks for learning and extracting static frame appearance information and motion timing information of videos, such as adding a temporal dimension to the 2D convolutional kernel of convolutional neural networks and expanding it to the 3D convolutional kernel to extract both spatial and temporal dimensional features. Considering the strong complementarity between spatial flow features and optical flow features, choosing a suitable fusion method can effectively improve the video classification performance. As a result, a recognition rate of almost 100% is obtained. When the trajectory feature is used, the trajectory information of the limb movement is captured, which greatly enhances the expression of behavior. Especially when the MBH descriptor is used, the recognition rate of boxing and applause is increased by more than 20%. The method first extracts video image frames to form image sequences and optical flow sequences, then extracts spatial flow features and optical flow features by the convolutional neural network, and introduces optical flow attention layer from temporal flow network to spatial flow network by mining the nearest neighbor relationship and association information between features in the flow embedded spatial flow convolutional neural network to guide the spatial flow to pay more attention to the human foreground region and reduce the influence of background noise. Thus, the variations and differences between spatial-temporal features are better obtained, as shown in Figure 3.

The visual attention mechanism is a unique signal processing mechanism of the human brain; through the observation of the global sample to determine the focus area and area of interest, the key information closely associated with the target will be quickly accessed; attention mechanism frees people from the colorful and complicated information and improves the efficiency of information processing, and it is introduced into the field of computer vision to improve the computer to solve image, video, and other prediction and analysis tasks. Consider that optical flow can be used to direct human foreground attention when appropriate compensation is applied to the movement of the lens. We investigate the combination of spatial streaming embedding CNN and temporal streaming CNN to form a dual-stream convolutional neural network to learn video features [20]. The purpose of introducing an optical flow attention layer from the temporal network to the spatial network is to guide the spatial flow to pay more attention to the human foreground region and to reduce the effect of background noise. Thus, the variation and differences between spatial-temporal features are better obtained and the rationality of the network to extract video features is improved. Attention is a mechanism used to give more weight to a subset of elements, and the optical flow attention map is directed to foreground regions and helps the spatial flow convolutional network to learn distributed feature representations around these regions to accomplish the label prediction task. In dual-stream convolutional networks, we propose an optical-stream attention layer to model the interaction of the two networks, which can be trained end-to-end using stochastic gradient descent and back-propagation algorithms.

Improve the efficiency of users to obtain information. In addition, the video semantic analysis technology based on the host computer can replace manual annotation work, reduce a lot of human resources, and improve the utilization rate of information. Considering the perceptual wildness of spatial information, the range of neighboring points can be expanded. When building a graph structure, the most extreme case, where the current node can be associated with all other nodes on the graph, can be achieved by the subsequent adoption of attention mechanisms or the amount of information passed. The inclusion of all nodes in the graph, as well as the interconnection of all nodes to each other, is a fully connected graph, constituting a complete graph that allows information about all locations to be perceived by each other. It allows each member to have a large enough perceptual field to recognize a larger range of spatial patterns.

## 4. Analysis of Results

### 4.1. Performance Results of High-Level Semantic Recognition Models under Engineering Management

By changing the length of the input sequence from 5 to 10 frames, the accuracy of the model was improved by 0.9%, but as we continued to increase the length of the input sequence to 15 and 20 frames, the accuracy of the model started to decrease. The reason for this phenomenon is that the size of the video dataset is relatively small and overfitting occurs when the input sequence is too long. Since each RGB image frame corresponds to 10 adjacent frames of the stacked optical flow image, the 10-frame input contains 100 consecutive frames of spatial-temporal information in the video clip, which is sufficient to represent the main semantic information of the video clip.

**Figure 4:**
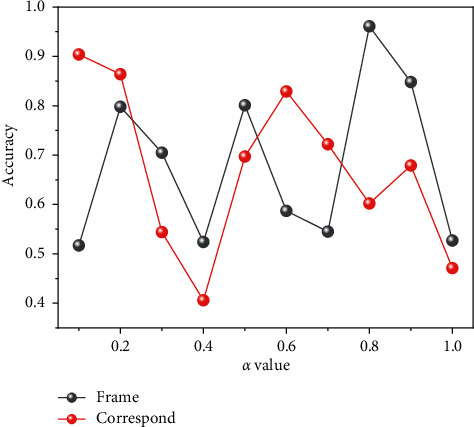
Effect of confidence parameters on model performance.

After the selection of the best input sequence is completed, the two-stream network stream embedding parameters and the confidence fusion parameters are set, and the experiments are firstly conducted to search the grid for the two parameters, and the best parameter for stream embedding is obtained as 0.2. After the stream embedding parameters are fixed, the experimental analysis of the effect of the confidence fusion parameter changes on the model performance is conducted, and the semantic concept detection accuracy based on different confidence fusion parameters is shown in Figure 4.

**Figure 5:**
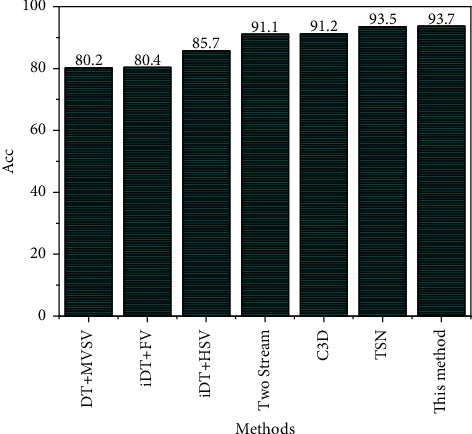
Results of different methods on the UCF-101 dataset.

There is difficulty in designing an efficient and robust identification method. Although the traditional manual labeling method can realize the understanding and description of the video semantic concept to a certain extent, the time and labor cost of manual labeling is huge, and the subjectivity is strong. The vertical coordinate in the Cartesian coordinate system indicates the corresponding video semantic detection accuracy at different confidence parameters. The video semantic concept detection accuracy keeps improving when the values are taken in the interval [0.1, 0.7], which proves that the confidence of the classifier based on the probability error has an important contribution to the final category prediction. The model prediction performance is best when it is 0.7, so this chapter chooses to take a value of 0.7 as the confidence parameter of the dual-stream network classifier. The performance of feature engineering-based algorithms IDT remains competitive; in addition, many methods based on deep learning were combined with IDT to achieve better results, but several video semantic analysis methods with the best model performance are deep convolutional network-based algorithms, and CD methods do not have an advantage over traditional methods due to many model parameters and more difficult training. The basic dual-stream network model has achieved good results by emulating the human visual mechanism and has a better understanding of the spatial and temporal information of the video. The TSN method is built based on the dual-stream network model, which can learn video features efficiently by modeling long time scales and combining sparse sampling strategies and video supervision methods, and it has achieved good results. The proposed method in this paper has 0.4% higher accuracy than TSN. This shows that the proposed method can better reflect the nearest neighbor relationship between samples and structural features, as well as the complementary relationship between images and optical flow, and the method of confidence fusion classification can effectively obtain video semantic concept features and improve the accuracy of video semantic concept detection, as shown in Figure 5.

The research has important academic significance and application value and helps to improve the level of video management technology, making it more complete and more efficient. In the process of optimal learning of video, features consider the nearest neighbor relationship between samples, association features, and so forth to construct stream shape constraint terms; optical flow attention mechanism was introduced to guide the spatial flow to pay more attention to the foreground region and reduce the influence of background noise, and, to better obtain the changes and differences between spatial-temporal features, in the acquisition of contextual information of video frame sequences, LSTM was introduced to construct stream shape embedding and optical flow attention based dual-stream CNN video semantic concept detection model. The proposed method can better reflect the nearest neighbor relationship and structural features between samples, as well as the complementary relationship between images and optical streams to obtain effective video semantic concept features, and the confidence fusion classification method for the category score results of the two-stream SoftMax layer can more effectively improve the accuracy of video semantic concept detection.

### 4.2. Experimental Results of Sports Video Content Analysis

**Figure 6:**
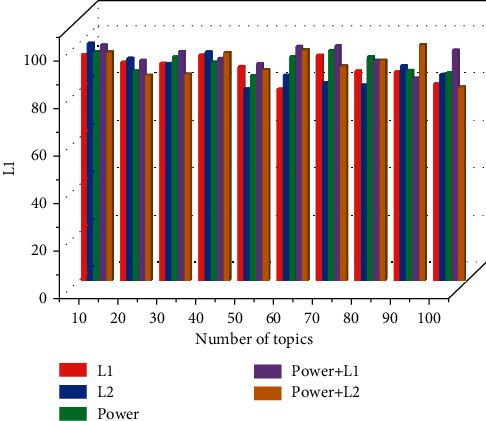
Performance comparison of different coding and normalization methods.

As shown in Figure 6, the classification accuracies achieved by different coding and normalization methods are compared using spatial-temporal interest point features with the number of topics varying between 10 and 100. As the number of topics increases, all coding methods achieve significant performance gains, but after the number of topics is 60, the performance does not change much. The difference between the results obtained when using vector quantization and local soft assignment is small, and the different normalization methods make a limited contribution to the recognition rate, with exponential plus l normalization achieving the best classification accuracy for most of the number of topics. The performance mostly improved as the number of topics increased, and the best performance was obtained when the number of topics reached 80 and then decreased. For soft assignment coding, there was a more significant decrease in classification performance compared to the results for spatial-temporal interest points, and the performance fluctuated by a maximum of more than 15 percentage points using different normalization methods. For both classes of descriptors, soft assignment coding tended to achieve optimal performance in combination with l. Group behavior recognition technology can derive tags of crowd scene images and can provide clues for retrieval of pictures and videos of group scenes. The advancement of group behavior recognition technology has greatly promoted the classification, labeling, and retrieval of crowd scenes.

**Figure 7:**
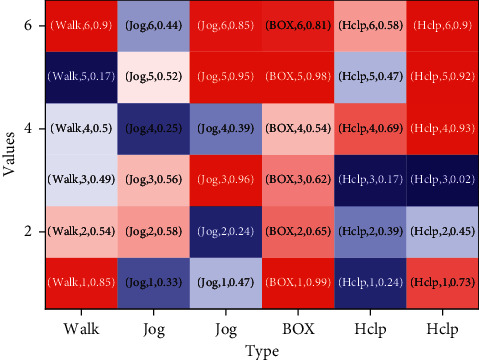
Content analysis results.

In Figure 7, the confusion matrix under different features is presented, and it is evident that walking and waving have the highest recognition rate among all cases, and, correspondingly, boxing and clapping have the lowest recognition rate. This is in line with the expectation that, in terms of form movements, boxing and clapping focused on upper limb movements and have a high degree of similarity, while walking and waving, which are more differentiated from the rest of the behaviors, obtain a recognition rate of nearly 100%. When trajectory features are used, the trajectory information of the limb movements is captured, which substantially enhances the representation of the behaviors, especially when MBH descriptors are used, increasing the recognition rate of boxing and clapping by more than 20 percentage points at maximum.

We obtained a classification accuracy of 89.63% using spatial-temporal interest points, which results in a 6-percentage point improvement. It is reasonable to assume that similar behaviors have similar characteristics and thematic distributions. Describing behaviors with mixed topic probability distributions is superior to the approach of corresponding a topic to a class of behaviors. One advantage of the topic model is that topics can be considered as a mid-level semantic feature and then used to describe more complex behaviors. Inevitably, there are similar form movements in different behaviors; for example, boxing and clapping both have similar upper body movements. Thus, different behaviors share the same themes, and each behavior has its distribution of themes, which enhances the discriminative nature of the features.

Overall, principal component analysis preprocessing of raw features not only reduces the feature dimensionality, thus making it less demanding on computational resources, but also retains most of the discriminative primary information, while having a suppressive effect on noise caused by various reasons, and whitening was also performed in the experiments to reduce the correlation between features, further improving the robust performance of recognition. The difference in the performance of the same action by different people; the influence of environmental factors, such as occlusion, viewing angle changes, lighting differences, and dynamic background interference, etc; and the start and end points of the action are blurred. The use of principal component analysis to preprocess raw features has an important impact on improving the performance of recognition. The principal component analysis projects the original features onto the feature components, which objectively suppresses the noise to a certain extent but, at the same time, inevitably brings about a loss of information. These two effects cancel each other out; if the noise component is large, the utility achieved by suppressing noise is large, which brings an increase in recognition rate, while the information loss effect is large and the corresponding performance is decreased. On the other hand, the performance of densely sampled features is superior, and the number of features to be processed is increasing, especially for video signals, which are particularly computationally intensive. If PCA is used to preprocess the original features, the number of feature dimensions is significantly reduced while retaining most of the information, resulting in little degradation in classification performance, which will greatly reduce the computational effort and improve the response speed, which is significant for applications that require real-time signal processing.

## 5. Conclusion

Better performance has been achieved after its introduction into the field of computer vision. In the word-packet framework, it has been shown that different feature encoding methods have an important impact on performance. Inspired by this, the impact of different coding methods combined with normalization methods on the classification performance of probabilistic implicit semantic analysis models is focused on, and it is found experimentally that local soft assignment coding combined with exponential normalization methods substantially improves the recognition performance; the impact of principal component analysis preprocessing raw features on performance is also examined, and when the features contain more noisy components, the computational effort is significantly reduced, while the classification recognition performance is even improved when the features contain more noisy components. However, the performance improvement of the fusion model for the language recognition model is limited. In addition, the idea of this paper is still stuck on the traditional pattern recognition task flow of the feature extraction-classification recognition model, and the two separated links may also affect the performance of the model to some extent. Therefore, a language recognition model based on an end-to-end approach is a very promising problem. Then the spatial flow features and optical flow features are extracted by the convolutional neural network, and the nearest neighbor relationship and association information between features are mined by stream shape embedded in spatial flow convolutional neural network, and the optical flow attention layer from the temporal flow network to spatial flow network is introduced to guide the spatial flow to pay more attention to the human foreground region and reduce the influence of background noise so that the variations and differences between spatial-temporal features can be better obtained. Then the features obtained from the two streams were input in temporal order to learn temporal features, and, finally, confidence fusion was performed on the classifier results of the two streams to detect the video semantic concept categories.

## Figures and Tables

**Table 1:** Network specific to each campaign.

Name	Ct-cn	Id-id	Kazak	Ko-kr	Tibet
Ct-cn	1233	11	0	23	0
Id-id	11	1234	11	0	0
Kazak	23	11	1234	11	23
Ko-kr	0	0	11	1234	11
Tibet	35	0	223	11	1234

## Data Availability

The data used to support the findings of this study are available upon request to the author.
